# 2-Fluoro-*N*′-[(2-meth­oxy­naphthalen-1-yl)methyl­idene]benzohydrazide

**DOI:** 10.1107/S1600536812011580

**Published:** 2012-03-24

**Authors:** He-Bing Li

**Affiliations:** a Department of Chemistry and Life Sciences, Xiangnan University, Chenzhou 423000, People’s Republic of China

## Abstract

The asymmetric unit of the title compound, C_19_H_15_FN_2_O_2_, contains two mol­ecules, *A* and *B*, in which the dihedral angles between the ring systems are 46.4 (2) and 17.24 (14)°, respectively. In the crystal, mol­ecules are linked into [010] chains of alternating *A* and *B* species by N—H⋯O hydrogen bonds.

## Related literature

For a related structure and background to hydrazones, see: Li (2011 ▶). For related structures, see: Qiu *et al.* (2006 ▶); Yang & Guo (2006 ▶); Yang (2006 ▶).
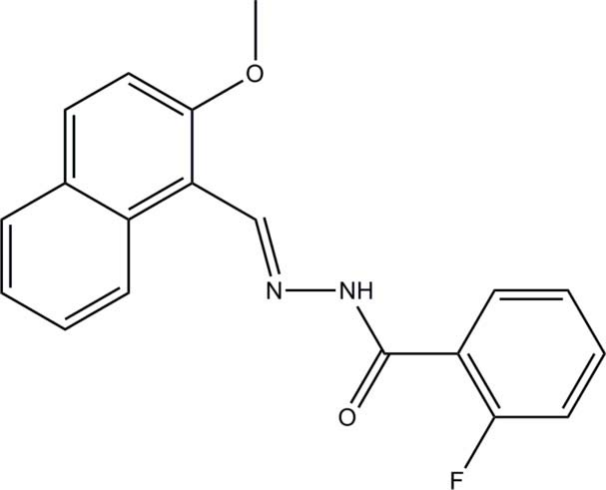


## Experimental

### 

#### Crystal data

C_19_H_15_FN_2_O_2_
*M* *_r_* = 322.33
Monoclinic, 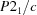

*a* = 9.462 (2) Å
*b* = 17.030 (3) Å
*c* = 20.470 (2) Å
β = 96.575 (2)°
*V* = 3276.9 (10) Å^3^
*Z* = 8
Mo *K*α radiation
μ = 0.09 mm^−1^
*T* = 298 K
0.18 × 0.18 × 0.17 mm

#### Data collection

Bruker SMART CCD diffractometer
Absorption correction: multi-scan (*SADABS*; Sheldrick, 1996 ▶) *T*_min_ = 0.983, *T*_max_ = 0.984
19271 measured reflections
6420 independent reflections
3070 reflections with *I* > 2σ(*I*)
*R*_int_ = 0.060

#### Refinement

*R*[*F* ^2^ > 2σ(*F* ^2^)] = 0.064
*wR*(*F* ^2^) = 0.188
*S* = 1.01
6420 reflections
441 parameters
2 restraints
H atoms treated by a mixture of independent and constrained refinement
Δρ_max_ = 0.53 e Å^−3^
Δρ_min_ = −0.30 e Å^−3^

### 

Data collection: *SMART* (Bruker, 1998 ▶); cell refinement: *SAINT* (Bruker, 1998 ▶); data reduction: *SAINT*; program(s) used to solve structure: *SHELXS97* (Sheldrick, 2008 ▶); program(s) used to refine structure: *SHELXL97* (Sheldrick, 2008 ▶); molecular graphics: *SHELXTL* (Sheldrick, 2008 ▶); software used to prepare material for publication: *SHELXTL*.

## Supplementary Material

Crystal structure: contains datablock(s) global, I. DOI: 10.1107/S1600536812011580/hb6672sup1.cif

Structure factors: contains datablock(s) I. DOI: 10.1107/S1600536812011580/hb6672Isup2.hkl

Supplementary material file. DOI: 10.1107/S1600536812011580/hb6672Isup3.cml

Additional supplementary materials:  crystallographic information; 3D view; checkCIF report

## Figures and Tables

**Table 1:** Hydrogen-bond geometry (Å, °)

*D*—H⋯*A*	*D*—H	H⋯*A*	*D*⋯*A*	*D*—H⋯*A*
N1—H1⋯O3^i^	0.90 (1)	2.02 (1)	2.901 (3)	166 (3)
N3—H3⋯O1^ii^	0.90 (1)	2.07 (2)	2.917 (3)	156 (3)

Symmetry codes: (i) 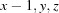; (ii) 
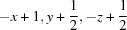
.

**Fig. 1.**
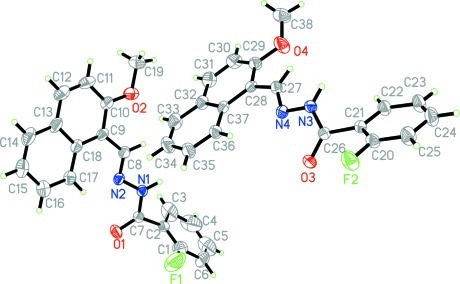
The molecular structure of the title compound, showing 30% probability displacement ellipsoids.

**Fig. 2.**
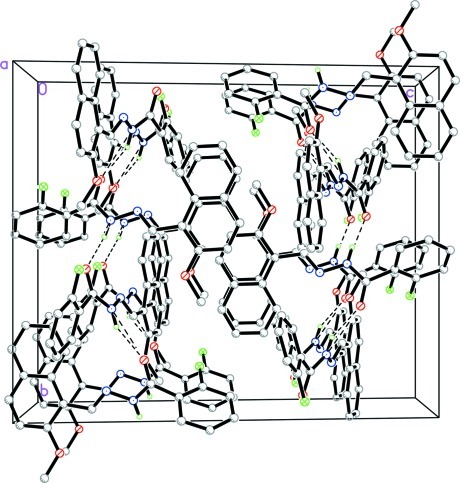
The crystal packing of the title compound. Hydrogen bonds are shown as dashed lines.

## supplementary crystallographic information

### Comment

As part of our ongoing studies of hydrazones (Li, 2011), the structure of the title compound is now reported.

The bond lengths and bond angles in the title compound (Fig. 1) are comprable with those observed in similar compounds (Qiu *et al.*, 2006; Yang & Guo, 2006; Yang, 2006). The dihedral angle between the C9—C18 naphthyl ring and C1—C1 benzene ring is 46.3 (2)° and the equivalent angle in the second molecule is 17.24 (14)°. In the crystal, the molecules are linked into chains along the *b* axis by N—H···O hydrogen bonds (Table 1 and Fig. 2).

### Experimental

2-Methoxy-1-naphthaldehyde (0.1 mmol, 18.6 mg) and 2-fluorobenzohydrazide (0.1 mmol, 12.4 mg) were dissolved in methanol (10 ml). The mixture was stirred at room temperature for 10 min to give a clear colorless solution. Colourless needles of the title compound were formed by gradual evaporation of the solvent over a week at room temperature (yield 78%).

### Refinement

Atoms H1 and H3 were located in a difference Fourier map and refined isotropically, with the N—H distance restrained to 0.90 (1) Å. The other H atoms of the compound were placed in geometrically idealized positions and allowed to ride on their parent atoms, with C—H = 0.93–0.96 Å, and with *U*_iso_(H) = 1.2*U*_eq_(C) and 1.5*U*_eq_(C19 and C38).

### Crystal data

**Table:**

C_19_H_15_FN_2_O_2_	*F*(000) = 1344
*M**_r_* = 322.33	*D*_x_ = 1.307 Mg m^−^^3^
Monoclinic, *P*2_1_/*c*	Mo *K*α radiation, λ = 0.71073 Å
Hall symbol: -P 2ybc	Cell parameters from 2255 reflections
*a* = 9.462 (2) Å	θ = 2.4–24.5°
*b* = 17.030 (3) Å	µ = 0.09 mm^−^^1^
*c* = 20.470 (2) Å	*T* = 298 K
β = 96.575 (2)°	Cut from needle, colorless
*V* = 3276.9 (10) Å^3^	0.18 × 0.18 × 0.17 mm
*Z* = 8	

### Data collection

**Table:**

Bruker SMART CCD diffractometer	6420 independent reflections
Radiation source: fine-focus sealed tube	3070 reflections with *I* > 2σ(*I*)
Graphite monochromator	*R*_int_ = 0.060
ω scans	θ_max_ = 26.0°, θ_min_ = 2.2°
Absorption correction: multi-scan (*SADABS*; Sheldrick, 1996)	*h* = −11→11
*T*_min_ = 0.983, *T*_max_ = 0.984	*k* = −20→20
19271 measured reflections	*l* = −18→25

### Refinement

**Table:**

Refinement on *F*^2^	Primary atom site location: structure-invariant direct methods
Least-squares matrix: full	Secondary atom site location: difference Fourier map
*R*[*F*^2^ > 2σ(*F*^2^)] = 0.064	Hydrogen site location: inferred from neighbouring sites
*wR*(*F*^2^) = 0.188	H atoms treated by a mixture of independent and constrained refinement
*S* = 1.01	*w* = 1/[σ^2^(*F*_o_^2^) + (0.0767*P*)^2^ + 0.7482*P*] where *P* = (*F*_o_^2^ + 2*F*_c_^2^)/3
6420 reflections	(Δ/σ)_max_ < 0.001
441 parameters	Δρ_max_ = 0.53 e Å^−^^3^
2 restraints	Δρ_min_ = −0.30 e Å^−^^3^

### Special details

**Table:**

Geometry. All e.s.d.'s (except the e.s.d. in the dihedral angle between two l.s. planes) are estimated using the full covariance matrix. The cell e.s.d.'s are taken into account individually in the estimation of e.s.d.'s in distances, angles and torsion angles; correlations between e.s.d.'s in cell parameters are only used when they are defined by crystal symmetry. An approximate (isotropic) treatment of cell e.s.d.'s is used for estimating e.s.d.'s involving l.s. planes.
Refinement. Refinement of *F*^2^ against ALL reflections. The weighted *R*-factor *wR* and goodness of fit *S* are based on *F*^2^, conventional *R*-factors *R* are based on *F*, with *F* set to zero for negative *F*^2^. The threshold expression of *F*^2^ > σ(*F*^2^) is used only for calculating *R*-factors(gt) *etc*. and is not relevant to the choice of reflections for refinement. *R*-factors based on *F*^2^ are statistically about twice as large as those based on *F*, and *R*- factors based on ALL data will be even larger.

### Fractional atomic coordinates and isotropic or equivalent isotropic displacement parameters (Å^2^)

**Table:**

	*x*	*y*	*z*	*U*_iso_*/*U*_eq_	
F1	−0.0022 (4)	0.3477 (2)	0.06984 (17)	0.1590 (14)	
F2	1.3946 (2)	0.57294 (11)	0.16914 (12)	0.0780 (7)	
H1	−0.008 (4)	0.4855 (11)	0.2092 (17)	0.080*	
H3	1.086 (4)	0.7327 (10)	0.2401 (18)	0.080*	
N1	−0.0394 (3)	0.43870 (15)	0.22248 (13)	0.0457 (7)	
N2	0.0044 (3)	0.41427 (15)	0.28601 (13)	0.0469 (7)	
N3	1.0565 (3)	0.68423 (14)	0.22805 (13)	0.0468 (7)	
N4	0.9273 (3)	0.65685 (15)	0.24414 (13)	0.0463 (7)	
O1	−0.1478 (3)	0.32290 (13)	0.19477 (12)	0.0626 (7)	
O2	0.1075 (3)	0.59232 (16)	0.39855 (13)	0.0798 (8)	
O3	1.0985 (2)	0.57402 (13)	0.17140 (11)	0.0580 (6)	
O4	0.7134 (3)	0.83626 (14)	0.29575 (15)	0.0837 (9)	
C1	−0.1047 (6)	0.3992 (3)	0.0618 (3)	0.0899 (14)	
C2	−0.1593 (4)	0.4240 (2)	0.11484 (17)	0.0554 (9)	
C3	−0.2676 (6)	0.4823 (3)	0.1082 (3)	0.1151 (19)	
H3A	−0.3091	0.5027	0.1436	0.138*	
C4	−0.3049 (8)	0.5056 (3)	0.0437 (4)	0.153 (3)	
H4	−0.3757	0.5434	0.0362	0.183*	
C5	−0.2457 (11)	0.4774 (4)	−0.0104 (4)	0.167 (4)	
H5	−0.2777	0.4956	−0.0524	0.200*	
C6	−0.1433 (8)	0.4244 (4)	−0.0018 (2)	0.132 (3)	
H6	−0.0995	0.4050	−0.0369	0.158*	
C7	−0.1146 (3)	0.38962 (19)	0.18080 (15)	0.0427 (8)	
C8	0.0679 (3)	0.4674 (2)	0.32235 (16)	0.0498 (8)	
H8	0.0799	0.5165	0.3039	0.060*	
C9	0.1224 (3)	0.4555 (2)	0.39083 (16)	0.0502 (9)	
C10	0.1416 (4)	0.5229 (2)	0.42919 (18)	0.0646 (10)	
C11	0.1938 (6)	0.5182 (3)	0.4958 (2)	0.1005 (16)	
H11	0.2067	0.5637	0.5208	0.121*	
C12	0.2255 (6)	0.4478 (3)	0.5238 (2)	0.1048 (17)	
H12	0.2594	0.4457	0.5683	0.126*	
C13	0.2088 (4)	0.3779 (3)	0.48811 (18)	0.0711 (11)	
C14	0.2415 (5)	0.3050 (3)	0.5180 (2)	0.0904 (14)	
H14	0.2767	0.3035	0.5623	0.109*	
C15	0.2231 (4)	0.2367 (3)	0.4841 (2)	0.0800 (13)	
H15	0.2438	0.1889	0.5049	0.096*	
C16	0.1727 (4)	0.2394 (2)	0.4175 (2)	0.0681 (11)	
H16	0.1596	0.1929	0.3938	0.082*	
C17	0.1425 (3)	0.3090 (2)	0.38666 (18)	0.0556 (9)	
H17	0.1114	0.3091	0.3419	0.067*	
C18	0.1572 (3)	0.3809 (2)	0.42067 (16)	0.0517 (9)	
C19	0.1272 (6)	0.6641 (2)	0.4349 (2)	0.1031 (16)	
H19A	0.0739	0.6621	0.4720	0.155*	
H19B	0.0947	0.7073	0.4071	0.155*	
H19C	0.2263	0.6708	0.4499	0.155*	
C20	1.3748 (4)	0.64862 (18)	0.15306 (17)	0.0503 (9)	
C21	1.2493 (3)	0.68480 (17)	0.16285 (15)	0.0407 (7)	
C22	1.2363 (4)	0.76311 (19)	0.14472 (17)	0.0535 (9)	
H22	1.1536	0.7902	0.1510	0.064*	
C23	1.3431 (4)	0.8014 (2)	0.11767 (19)	0.0644 (10)	
H23	1.3308	0.8534	0.1046	0.077*	
C24	1.4673 (4)	0.7636 (2)	0.1098 (2)	0.0735 (12)	
H24	1.5400	0.7901	0.0921	0.088*	
C25	1.4845 (4)	0.6864 (2)	0.1280 (2)	0.0708 (11)	
H25	1.5690	0.6601	0.1233	0.085*	
C26	1.1294 (3)	0.64171 (18)	0.18800 (15)	0.0424 (8)	
C27	0.8576 (3)	0.70852 (19)	0.27226 (15)	0.0461 (8)	
H27	0.9017	0.7568	0.2811	0.055*	
C28	0.7151 (3)	0.69900 (18)	0.29183 (14)	0.0419 (7)	
C29	0.6442 (4)	0.7680 (2)	0.30429 (17)	0.0538 (9)	
C30	0.5059 (4)	0.7667 (2)	0.32251 (19)	0.0678 (11)	
H30	0.4593	0.8136	0.3295	0.081*	
C31	0.4411 (4)	0.6978 (2)	0.32980 (19)	0.0681 (11)	
H31	0.3492	0.6976	0.3418	0.082*	
C32	0.5078 (3)	0.6258 (2)	0.31977 (17)	0.0549 (9)	
C33	0.4409 (4)	0.5537 (3)	0.3293 (2)	0.0742 (12)	
H33	0.3506	0.5536	0.3431	0.089*	
C34	0.5048 (4)	0.4843 (2)	0.3190 (2)	0.0772 (12)	
H34	0.4589	0.4372	0.3257	0.093*	
C35	0.6399 (4)	0.4842 (2)	0.29817 (19)	0.0663 (10)	
H35	0.6836	0.4367	0.2904	0.080*	
C36	0.7081 (4)	0.55235 (18)	0.28913 (17)	0.0534 (9)	
H36	0.7987	0.5508	0.2757	0.064*	
C37	0.6458 (3)	0.62534 (18)	0.29945 (14)	0.0422 (8)	
C38	0.6786 (5)	0.9035 (2)	0.3292 (2)	0.0879 (14)	
H38A	0.5837	0.9197	0.3132	0.132*	
H38B	0.7442	0.9447	0.3220	0.132*	
H38C	0.6838	0.8923	0.3754	0.132*	

### Atomic displacement parameters (Å^2^)

**Table:**

	*U*^11^	*U*^22^	*U*^33^	*U*^12^	*U*^13^	*U*^23^
F1	0.181 (4)	0.188 (3)	0.119 (3)	0.030 (3)	0.062 (3)	−0.018 (2)
F2	0.0723 (14)	0.0502 (13)	0.1137 (19)	0.0092 (10)	0.0203 (13)	0.0088 (12)
N1	0.0481 (16)	0.0476 (17)	0.0404 (16)	−0.0057 (13)	0.0003 (13)	0.0044 (14)
N2	0.0487 (16)	0.0549 (17)	0.0366 (16)	−0.0028 (13)	0.0026 (13)	0.0052 (14)
N3	0.0456 (16)	0.0439 (16)	0.0534 (17)	−0.0090 (13)	0.0170 (13)	−0.0083 (14)
N4	0.0436 (16)	0.0489 (16)	0.0489 (17)	−0.0040 (13)	0.0160 (13)	−0.0021 (13)
O1	0.0756 (17)	0.0448 (14)	0.0638 (16)	−0.0116 (12)	−0.0074 (13)	0.0118 (12)
O2	0.112 (2)	0.0629 (18)	0.0635 (18)	−0.0032 (15)	0.0045 (16)	−0.0105 (15)
O3	0.0672 (15)	0.0424 (14)	0.0685 (16)	−0.0146 (11)	0.0257 (13)	−0.0102 (12)
O4	0.0845 (19)	0.0444 (15)	0.130 (3)	0.0030 (14)	0.0466 (18)	−0.0075 (15)
C1	0.106 (4)	0.081 (3)	0.082 (4)	0.007 (3)	0.008 (3)	0.003 (3)
C2	0.075 (2)	0.050 (2)	0.039 (2)	−0.0166 (19)	−0.0042 (19)	−0.0007 (17)
C3	0.131 (4)	0.092 (3)	0.105 (4)	0.018 (3)	−0.061 (3)	0.053 (3)
C4	0.221 (8)	0.092 (4)	0.125 (5)	0.026 (4)	−0.071 (6)	0.010 (4)
C5	0.278 (11)	0.105 (6)	0.092 (5)	−0.049 (6)	−0.088 (6)	0.040 (4)
C6	0.221 (8)	0.138 (6)	0.035 (3)	−0.055 (5)	0.008 (4)	0.001 (3)
C7	0.0421 (18)	0.0409 (19)	0.044 (2)	0.0030 (15)	0.0016 (15)	0.0016 (16)
C8	0.049 (2)	0.055 (2)	0.045 (2)	−0.0051 (17)	0.0051 (16)	0.0006 (17)
C9	0.0438 (19)	0.069 (2)	0.038 (2)	−0.0102 (17)	0.0039 (15)	−0.0053 (18)
C10	0.079 (3)	0.071 (3)	0.044 (2)	−0.012 (2)	0.0066 (19)	−0.003 (2)
C11	0.154 (5)	0.097 (4)	0.047 (3)	−0.019 (3)	−0.005 (3)	−0.019 (3)
C12	0.161 (5)	0.105 (4)	0.044 (3)	−0.014 (4)	−0.012 (3)	0.000 (3)
C13	0.086 (3)	0.088 (3)	0.038 (2)	−0.007 (2)	0.001 (2)	0.010 (2)
C14	0.109 (4)	0.111 (4)	0.050 (3)	0.001 (3)	0.002 (2)	0.020 (3)
C15	0.077 (3)	0.092 (3)	0.072 (3)	0.008 (2)	0.012 (2)	0.029 (3)
C16	0.053 (2)	0.074 (3)	0.077 (3)	0.0002 (19)	0.005 (2)	0.014 (2)
C17	0.049 (2)	0.065 (2)	0.052 (2)	−0.0016 (18)	0.0004 (17)	0.008 (2)
C18	0.0451 (19)	0.069 (3)	0.041 (2)	−0.0073 (17)	0.0056 (15)	0.0037 (19)
C19	0.145 (5)	0.071 (3)	0.098 (4)	−0.012 (3)	0.033 (3)	−0.027 (3)
C20	0.055 (2)	0.0336 (19)	0.063 (2)	0.0042 (16)	0.0136 (18)	0.0009 (16)
C21	0.0411 (18)	0.0403 (19)	0.0413 (18)	−0.0021 (14)	0.0076 (14)	−0.0017 (15)
C22	0.054 (2)	0.045 (2)	0.064 (2)	0.0011 (16)	0.0148 (18)	0.0060 (17)
C23	0.065 (2)	0.049 (2)	0.082 (3)	−0.0026 (19)	0.018 (2)	0.0143 (19)
C24	0.062 (3)	0.061 (3)	0.104 (3)	−0.008 (2)	0.039 (2)	0.006 (2)
C25	0.052 (2)	0.065 (3)	0.101 (3)	0.0006 (19)	0.032 (2)	0.000 (2)
C26	0.0468 (19)	0.0384 (19)	0.0424 (19)	−0.0009 (15)	0.0059 (15)	0.0013 (15)
C27	0.0460 (19)	0.0466 (19)	0.046 (2)	−0.0071 (16)	0.0083 (16)	0.0024 (16)
C28	0.0410 (18)	0.0499 (19)	0.0355 (17)	−0.0009 (15)	0.0079 (14)	−0.0034 (15)
C29	0.055 (2)	0.048 (2)	0.060 (2)	0.0018 (18)	0.0138 (18)	−0.0034 (18)
C30	0.057 (2)	0.064 (3)	0.084 (3)	0.017 (2)	0.017 (2)	−0.007 (2)
C31	0.047 (2)	0.081 (3)	0.079 (3)	0.004 (2)	0.020 (2)	−0.002 (2)
C32	0.043 (2)	0.066 (2)	0.057 (2)	−0.0038 (18)	0.0125 (16)	0.0014 (19)
C33	0.053 (2)	0.091 (3)	0.082 (3)	−0.012 (2)	0.023 (2)	0.003 (2)
C34	0.073 (3)	0.068 (3)	0.094 (3)	−0.024 (2)	0.023 (2)	0.003 (2)
C35	0.071 (3)	0.055 (2)	0.076 (3)	−0.006 (2)	0.023 (2)	0.002 (2)
C36	0.054 (2)	0.048 (2)	0.060 (2)	−0.0031 (17)	0.0193 (18)	−0.0012 (17)
C37	0.0404 (18)	0.051 (2)	0.0354 (18)	−0.0016 (15)	0.0047 (14)	0.0019 (15)
C38	0.107 (4)	0.054 (2)	0.104 (4)	0.006 (2)	0.016 (3)	−0.015 (2)

### Geometric parameters (Å, º)

**Table:**

F1—C1	1.304 (5)	C15—H15	0.9300
F2—C20	1.338 (3)	C16—C17	1.357 (5)
N1—C7	1.340 (4)	C16—H16	0.9300
N1—N2	1.383 (3)	C17—C18	1.407 (5)
N1—H1	0.903 (10)	C17—H17	0.9300
N2—C8	1.277 (4)	C19—H19A	0.9600
N3—C26	1.342 (4)	C19—H19B	0.9600
N3—N4	1.383 (3)	C19—H19C	0.9600
N3—H3	0.897 (10)	C20—C25	1.370 (5)
N4—C27	1.276 (4)	C20—C21	1.374 (4)
O1—C7	1.221 (3)	C21—C22	1.386 (4)
O2—C10	1.359 (4)	C21—C26	1.491 (4)
O2—C19	1.431 (4)	C22—C23	1.371 (4)
O3—C26	1.228 (3)	C22—H22	0.9300
O4—C29	1.356 (4)	C23—C24	1.366 (5)
O4—C38	1.392 (4)	C23—H23	0.9300
C1—C2	1.325 (6)	C24—C25	1.371 (5)
C1—C6	1.379 (7)	C24—H24	0.9300
C2—C3	1.421 (6)	C25—H25	0.9300
C2—C7	1.488 (4)	C27—C28	1.458 (4)
C3—C4	1.385 (8)	C27—H27	0.9300
C3—H3A	0.9300	C28—C29	1.390 (4)
C4—C5	1.383 (10)	C28—C37	1.432 (4)
C4—H4	0.9300	C29—C30	1.402 (5)
C5—C6	1.321 (9)	C30—C31	1.342 (5)
C5—H5	0.9300	C30—H30	0.9300
C6—H6	0.9300	C31—C32	1.404 (5)
C8—C9	1.451 (4)	C31—H31	0.9300
C8—H8	0.9300	C32—C33	1.406 (5)
C9—C10	1.392 (5)	C32—C37	1.415 (4)
C9—C18	1.431 (5)	C33—C34	1.355 (5)
C10—C11	1.398 (5)	C33—H33	0.9300
C11—C12	1.348 (6)	C34—C35	1.393 (5)
C11—H11	0.9300	C34—H34	0.9300
C12—C13	1.396 (6)	C35—C36	1.351 (4)
C12—H12	0.9300	C35—H35	0.9300
C13—C14	1.403 (5)	C36—C37	1.402 (4)
C13—C18	1.412 (5)	C36—H36	0.9300
C14—C15	1.355 (6)	C38—H38A	0.9600
C14—H14	0.9300	C38—H38B	0.9600
C15—C16	1.392 (6)	C38—H38C	0.9600
			
C7—N1—N2	119.4 (3)	O2—C19—H19B	109.5
C7—N1—H1	122 (2)	H19A—C19—H19B	109.5
N2—N1—H1	118 (2)	O2—C19—H19C	109.5
C8—N2—N1	113.9 (3)	H19A—C19—H19C	109.5
C26—N3—N4	119.6 (2)	H19B—C19—H19C	109.5
C26—N3—H3	120 (2)	F2—C20—C25	117.0 (3)
N4—N3—H3	120 (2)	F2—C20—C21	119.7 (3)
C27—N4—N3	113.1 (3)	C25—C20—C21	123.3 (3)
C10—O2—C19	119.5 (3)	C20—C21—C22	116.4 (3)
C29—O4—C38	119.8 (3)	C20—C21—C26	122.3 (3)
F1—C1—C2	117.7 (4)	C22—C21—C26	121.2 (3)
F1—C1—C6	116.3 (6)	C23—C22—C21	121.2 (3)
C2—C1—C6	126.0 (5)	C23—C22—H22	119.4
C1—C2—C3	119.3 (4)	C21—C22—H22	119.4
C1—C2—C7	121.4 (4)	C24—C23—C22	120.5 (3)
C3—C2—C7	119.2 (4)	C24—C23—H23	119.7
C4—C3—C2	113.2 (6)	C22—C23—H23	119.7
C4—C3—H3A	123.4	C23—C24—C25	119.8 (3)
C2—C3—H3A	123.4	C23—C24—H24	120.1
C5—C4—C3	125.6 (7)	C25—C24—H24	120.1
C5—C4—H4	117.2	C20—C25—C24	118.7 (3)
C3—C4—H4	117.2	C20—C25—H25	120.6
C6—C5—C4	119.2 (6)	C24—C25—H25	120.6
C6—C5—H5	120.4	O3—C26—N3	123.6 (3)
C4—C5—H5	120.4	O3—C26—C21	122.2 (3)
C5—C6—C1	116.8 (6)	N3—C26—C21	114.2 (3)
C5—C6—H6	121.6	N4—C27—C28	126.1 (3)
C1—C6—H6	121.6	N4—C27—H27	116.9
O1—C7—N1	124.3 (3)	C28—C27—H27	116.9
O1—C7—C2	121.7 (3)	C29—C28—C37	118.9 (3)
N1—C7—C2	114.0 (3)	C29—C28—C27	115.9 (3)
N2—C8—C9	123.7 (3)	C37—C28—C27	125.2 (3)
N2—C8—H8	118.1	O4—C29—C28	116.8 (3)
C9—C8—H8	118.1	O4—C29—C30	121.7 (3)
C10—C9—C18	118.9 (3)	C28—C29—C30	121.4 (3)
C10—C9—C8	116.0 (3)	C31—C30—C29	119.7 (3)
C18—C9—C8	125.1 (3)	C31—C30—H30	120.1
O2—C10—C9	116.7 (3)	C29—C30—H30	120.1
O2—C10—C11	122.6 (4)	C30—C31—C32	121.9 (3)
C9—C10—C11	120.7 (4)	C30—C31—H31	119.0
C12—C11—C10	120.2 (4)	C32—C31—H31	119.0
C12—C11—H11	119.9	C31—C32—C33	121.7 (3)
C10—C11—H11	119.9	C31—C32—C37	119.6 (3)
C11—C12—C13	121.9 (4)	C33—C32—C37	118.7 (3)
C11—C12—H12	119.0	C34—C33—C32	121.6 (3)
C13—C12—H12	119.0	C34—C33—H33	119.2
C12—C13—C14	121.3 (4)	C32—C33—H33	119.2
C12—C13—C18	119.2 (4)	C33—C34—C35	119.4 (4)
C14—C13—C18	119.5 (4)	C33—C34—H34	120.3
C15—C14—C13	121.9 (4)	C35—C34—H34	120.3
C15—C14—H14	119.1	C36—C35—C34	120.7 (4)
C13—C14—H14	119.1	C36—C35—H35	119.7
C14—C15—C16	118.8 (4)	C34—C35—H35	119.7
C14—C15—H15	120.6	C35—C36—C37	121.7 (3)
C16—C15—H15	120.6	C35—C36—H36	119.2
C17—C16—C15	121.0 (4)	C37—C36—H36	119.2
C17—C16—H16	119.5	C36—C37—C32	117.9 (3)
C15—C16—H16	119.5	C36—C37—C28	123.7 (3)
C16—C17—C18	121.7 (3)	C32—C37—C28	118.4 (3)
C16—C17—H17	119.2	O4—C38—H38A	109.5
C18—C17—H17	119.2	O4—C38—H38B	109.5
C17—C18—C13	117.1 (3)	H38A—C38—H38B	109.5
C17—C18—C9	123.8 (3)	O4—C38—H38C	109.5
C13—C18—C9	119.1 (3)	H38A—C38—H38C	109.5
O2—C19—H19A	109.5	H38B—C38—H38C	109.5

### Hydrogen-bond geometry (Å, º)

**Table:**

*D*—H···*A*	*D*—H	H···*A*	*D*···*A*	*D*—H···*A*
N1—H1···O3^i^	0.90 (1)	2.02 (1)	2.901 (3)	166 (3)
N3—H3···O1^ii^	0.90 (1)	2.07 (2)	2.917 (3)	156 (3)

Symmetry codes: (i) *x*−1, *y*, *z*; (ii) −*x*+1, *y*+1/2, −*z*+1/2.
